# Post-transcriptional modulation of cytochrome P450s, *Cyp6g1* and *Cyp6g2*, by *miR-310s* cluster is associated with DDT-resistant *Drosophila melanogaster* strain *91*-*R*

**DOI:** 10.1038/s41598-020-71250-0

**Published:** 2020-09-01

**Authors:** Keon Mook Seong, Brad S. Coates, Barry R. Pittendrigh

**Affiliations:** 1 Department of Applied Biology, College of Ecology and Environment, Kyungpook National University, Sangju, Korea; 2 USDA-ARS, Corn Insects and Crop Genetics Research Unit, Ames, IA USA; 3 Department of Entomology, Michigan State University, East Lansing, MI USA

**Keywords:** Molecular biology, Molecular evolution

## Abstract

The role of miRNAs in mediating insecticide resistance remains largely unknown, even for the model species *Drosophila melanogaster*. Building on prior research, this study used microinjection of synthetic *miR-310s* mimics into DDT-resistant *91-R* flies and observed both a significant transcriptional repression of computationally-predicted endogenous target P450 detoxification genes, *Cyp6g1* and *Cyp6g2*, and also a concomitant increase in DDT susceptibility. Additionally, co-transfection of *D. melanogaster* S2 cells with dual luciferase reporter constructs validated predictions that *miR-310s* bind to target binding sites in the 3ʹ untranslated regions (3ʹ-UTR) of both *Cyp6g1* and *Cyp6g2* in vitro. Findings in the current study provide empirical evidence for a link between reduced miRNA expression and an insecticidal resistance phenotype through reduced targeted post-transcriptional suppression of transcripts encoding proteins involved in xenobiotic detoxification. These insights are important for understanding the breadth of adaptive molecular changes that have contributed to the evolution of DDT resistance in *D. melanogaster*.

## Introduction

The exposure of a species to changing environmental conditions, such as variation in nutrient availability, climate, and toxic chemicals, can lead to corresponding phenotypic change(s) via adaptive directional selection. Insecticidal compounds—including synthetic chemicals, natural products, and protein toxins—represent human-imposed selection pressures upon insect populations. Specifically, insecticides have been widely used to suppress insect populations in efforts to protect human health by stabilizing the output of agricultural commodities and foodstuffs^1^, and reducing the incidence of insect vector-borne diseases, such as malaria and dengue fever^2,3^. However, frequent and widespread application of insecticides has contributed to the development of insect populations with high frequencies of phenotypic resistance to one or more classes of insecticides^4^. Such responses by insect populations and selection for high levels of resistance represent serious threats to many pest control programs. The evolution of insecticide resistance in insect populations involves genomic variations in the genome that, in turn, offers the scientific community an opportunity to both understand the genes directly involved in resistance and, in some cases, regulatory mechanisms associated with those genes. A model system that affords the opportunity to perform gene-by-gene analysis of traits involved in polygenic pesticide resistance is that of dichlorodiphenyltrichloroethane (DDT) resistance in *Drosophila melanogaster* (hereafter referred to as *Drosophila*).

DDT is an organochlorine insecticide that disrupts the insect nervous system by affecting the permeability of nerve cell plasma membranes and causing paralysis^5^. While DDT was extensively used during the post-World War II to control insect pests, deleterious side effects on non-target mammalian, bird, and insect species ultimately led to its ban by most countries by the 1980s^6^. Although DDT is no longer extensively used, selected laboratory colonies of *Drosophila* with varying levels of DDT resistance provide a model system for investigating adaptive genomic responses that lead to insecticide resistance^7^. The *Drosophila* model laboratory strain for DDT resistance, *91-R*, has been under chronic exposure to DDT for over six decades and reared in parallel with the non-selected control strain *91-C*; the two strains came from a common population that was split before these decades-long difference in treatment of the two populations. This high-level DDT resistance phenotype in *Drosophila* is polygenic and associated with multimodal resistance mechanisms^8,9^ including, but not limited to, involvement of phase I, II, and III detoxification enzymes. For example, the variance in protein structure and transcript expression of cytochrome P450 monooxygenases (P450s), including *Cyp6g1* and ATP-binding cassette (ABC) transporters, has been reported in the DDT-resistant *91-R* compared to DDT-susceptible strains *91-C* and *Canton-S*^10–13^. Additionally, directional selection was predicted to contribute to selective sweeps proposed within multiple genome regions of *91-R* compared to *91-C*^14^; among these implicated genes, the role of the ABC transporter, multidrug resistant (MDR) 49, in DDT resistance was validated using a transgenic expression approach^13^. Moreover, the involvement of several MDR and P450 genes were implicated in DDT resistance of *91-R* using transgenic knockdown lines^10^. Despite the implication of directional selection within multiple genome regions, the independent roles or additive/non-additive contributions to the DDT resistance phenotype in *91-R* remains unknown.

Among different genetic mechanisms implicated within DDT resistant phenotypes, P450s play pivotal roles in detoxifying exogenous xenobiotics such as insecticides and plant toxins through catabolic pathways that relegate compounds into more soluble and less toxic products^15^. Two possible mechanisms of P450-mediated insecticide resistance have been demonstrated. First, genome-wide association studies identified DDT-associated multiple genes and signatures of adaptive selection within the genome, where the amino acid changes in *Cyp6w1* were associated with DDT resistance^16^. Second, the increased abundance of transcripts for P450s and the likely increased (subsequent) levels of functional translated P450 enzymes have been proposed as a mechanism of resistance to several classes of insecticides^17^, including DDT in *Drosophila*^18–20^. For example, the potential involvement of *Cyp4g1*, *Cyp6g1*, and *Cyp12d1* in DDT resistance in *91-R* was demonstrated via independent RNA interface (RNAi)-mediated knockdown of transcripts using the transgenic Gal4/UAS-RNAi expression system, each resulting in increased susceptibility^10^. Moreover, differences in micro-RNA (miRNA) repression of P450 transcription or translation was implicated within the polygenic DDT resistance mechanism of *91-R*^11,21^. However, relatively little is known regarding the precise mechanism(s) by which P450s are silenced by miRNAs or the downstream effects on response to xenobiotic exposure in *Drosophila*.

miRNAs are small endogenous non-protein coding RNAs with lengths between 19 and 23 nucleotides. They are involved in many biological processes, including regulation of cellular metabolism and organismal homeostasis^22^. A given miRNA can negatively regulate the translation of mRNA transcripts by reverse complementarily binding the 3′-UTR of the target mRNA, which leads to post-transcriptional degradation of the mRNA^23^. This mRNA decay is preceded by miRNA-mediated inhibition of translation, indicating that transcriptional and translational silencing are intertwined^24^. Thus, miRNAs have important roles in regulating transcript levels for genes involved in insect development, behaviour, and host–pathogen interactions^23,25^. To date, there are few instances in which miRNAs are known to regulate genes that mediate insecticide resistance within insect species. One study example in the cotton aphid, *Aphis gossypii*, demonstrated that two miRNAs, *miR-276* and *miR-3016*, contribute to a spirotetramat resistance phenotype through the targeting of an acetyl-coenzyme A carboxylase gene^26^. Another study was associated with the down-regulation of the *miR-2*~*13*~*71* cluster in deltamethrin-resistant adult *Culex pipiens* with the concomitant increase in transcript levels of putative targets *Cyp325bg3* and *Cyp9j35* mRNAs^27^. In *Tetranychus cinnabarinus*, *tci-miR-1-3p* plays a critical role in cyflumetofen resistance by targeting a GST gene, TCGSTM4^28^.

In previous research, we identified miRNAs with significant levels of differential expression between *Drosophila* strains that are DDT-resistant (*91-R*) and -susceptible (*91-C*)^21^. The *miR-310s* were significantly down-regulated in the *91-R* strain as compared to the *91-C* strain and computational predictions identified a number of cytochrome P450 transcripts, including *Cyp6g1*, *Cyp6g2*, *Cyp6w1*, *Cyp49a1*, and *Cyp12a5,* as potential targets of these *miR-310s*^21^. However, empirical evidence to support these predicted regulatory roles of *miR-310s* in DDT resistance is currently lacking.

In the present study, we tested the hypothesis that *miR-310s* impact the levels of endogenous *Cyp6g1* and *Cyp6g2* transcripts in vivo and are associated with levels of DDT-resistance in adults for *91-R*. Furthermore, reporter assay experiments allowed us to test the hypothesis that computationally-predicted *miR-310s* seed regions in *Cyp6g1* and *Cyp6g2* 3ʹ -UTRs are linked to decreased levels of corresponding transcripts when co-expressed with *miR-310s*.

## Results

### Constitutive expression levels of target *Cyp6g1* and *Cyp6g2* genes of* miR-31*0s

The association between the up-regulation of *Cyp6g1* and *Cyp6g2* expression with DDT resistance in *91-R* was validated via comparison of RT-qPCR assay results with DDT–susceptible strains *91-C* and *Canton-S*. Specifically, our results showed a significantly higher level of constitutive *Cyp6g1* and *Cyp6g2* transcript expression in *91-R* as compared to both *91-C* and *Canton-S* (Supplemental Fig. S1; *F* = 154.8, df = 2, *p* < 0.05 for *Cyp6g1*; *F* = 93.7, df = 2, *p* < 0.05 for *Cyp6g2*). In contrast, neither *Cyp6g1* or *Cyp6g2* showed any significant level of differential expression between the DDT-susceptible strains *91-C* and *Canton-S* (*p* = 0.139 for *Cyp6g1*; *p* = 0.438 for *Cyp6g2*).

### Microinjection of *miR-31*0s impacts P450 gene regulation

**Figure 1:**
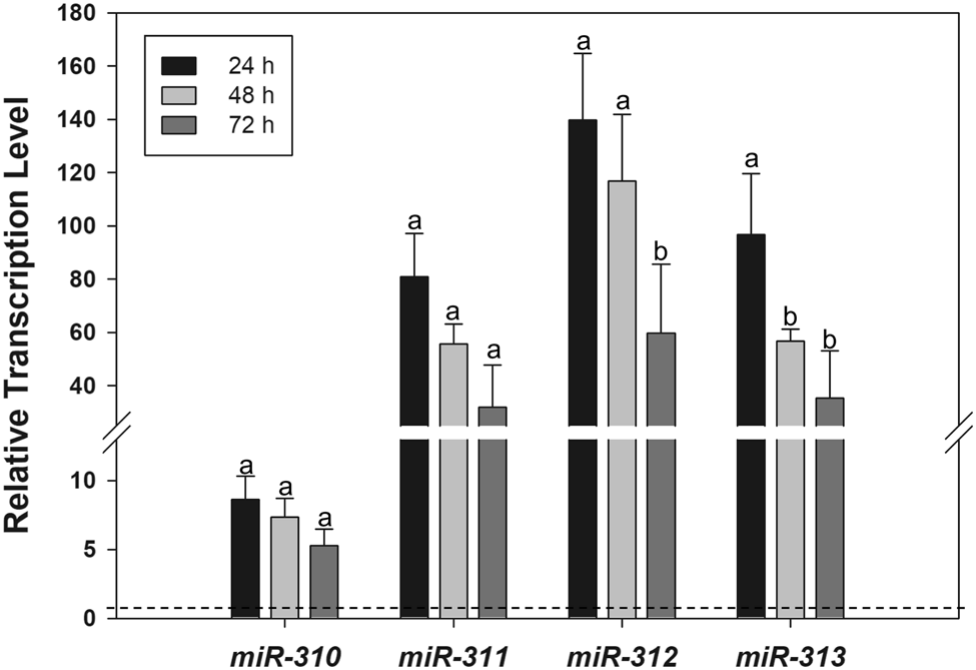
Relative transcription levels of *miR-310*, *miR-311*, *miR-312*, and *miR-313* after microinjection of the *miR-310s* mimics, negative control siRNA (NCsiRNA) mimic, and DEPC-water into *91-R* female at 24 h, 48 h, and 72 h post-injection. All levels of *miR-310s* are given relative to the transcription levels of NC and DEPC-water which were ascribed an arbitrary value of 1 (dashed line). There was no difference between the DEPC and NCsiRNA microinjection groups (*p* = 0.229–0.943). Different letters on the bars indicate that the means are significantly different across three time points within each miRNA (*p* < 0.05).

miRNA mimics are small, chemically modified double-stranded RNAs that mimic endogenous miRNAs. The microinjection of mimics for the *miR-310s* cluster (*miR-310*, *miR-311*, *miR-312*, and *miR-313*) was used to evaluate their potential role in mediating the regulation of putatively targeted cytochrome P450s-*Cyp6g1* and -*Cyp6g2* transcript levels in the *91-R* strain. Following the injection of *miR-310s* mimics into adult females of the *91-R* strain, the levels of these aforementioned miRNAs increased at all time points compared to the NCsiRNA and DEPC-injected control (Fig. 1). Specifically, temporal sampling showed that the increase in cellular *miR-310* levels were 8.7-, 7.4-, and 5.3-fold (*F* = 18.6, df = 4, *p* < 0.05) and cellular *miR-311* levels were 80.9-, 55.7-, and 31.9-fold (*F* = 22.4, df = 4, *p* < 0.05) as compared to the controls at 24 h, 48 h, and 72 h following mimic injections, respectively (Fig. 1). However, the relative increase in cellular *miR-310* and *miR-311* mimic levels were not significantly different across the three time points. When *miR-312* and *miR-313* mimic levels were measured, their increases occurred with estimates of 139.8-, 117-, 59.7-fold (F = 37.8, df = 4, *p* < 0.05) for *miR-312* mimic and 96.8-, 56.7-, and 35.4-fold (F = 80.2, df = 4, *p* < 0.05) for *miR-313* mimic at 24 h, 48 h, and 72 h post-injection as compared to the controls (Fig. 1). The relative increases in cellular *miR-312* and *miR-313* mimic levels were the greatest at 24 h as compared to the controls (*p* < 0.05; Fig. 1). When measured 72 h after injection, however, the levels of *miR-312* and *miR-313* mimics had significantly declined as compared to 24 h post-injection interval (*p* < 0.05; Fig. 1).

**Figure 2:**
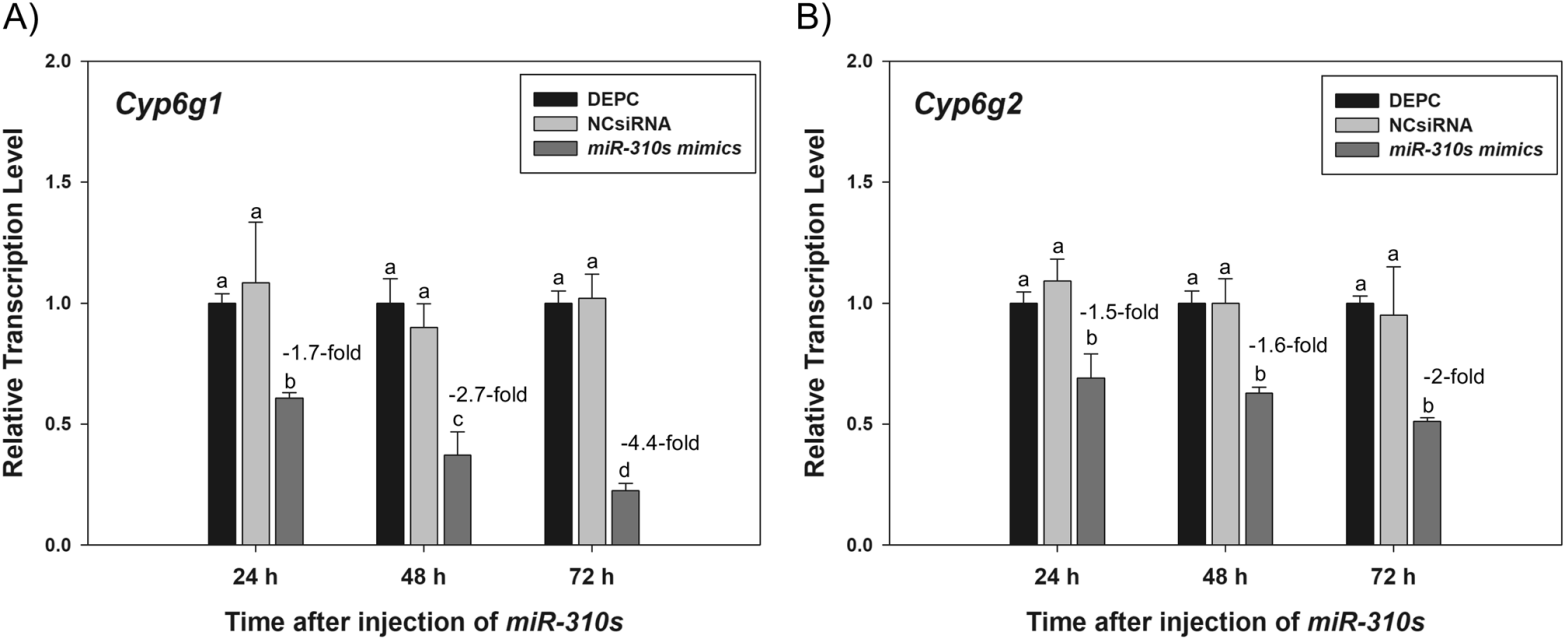
Relative transcription levels of (**A**) *Cyp6g1* and (**B**) *Cyp6g2* after microinjection of the *miR-310s* mimics, negative control siRNA (NCsiRNA) mimic, and DEPC-water into *91-R* female flies at 24 h, 48 h, and 72 h post-injection. There was no difference between the DEPC-water and NC microinjection groups across three time points for *Cyp6g1* (*p* = 0.55) and *Cyp6g2* (*p* = 0.953). Different letters on the bars indicated that the means were significantly different across three time points (*p* < 0.05).

The cellular levels among the putatively targeted endogenous *Cyp6g1* and *Cyp6g2* transcripts were evaluated in response to *miR-310s* mimics injections in *91-R* strain. Specifically, flies in the *miR-310s* mimics-injected treatment groups showed a reduction in the levels of *Cyp6g1* and *Cyp6g2* transcripts as compared to NCsiRNA and DEPC-injected control (Fig. 2). The relative proportion of reduction in *Cyp6g1* transcript levels were 1.7-, 2.7-, and 4.4-fold at 24 h, 48 h, and 72 h after mimics injections, respectively (*F* = 73.4, df = 8, *p* < 0.05 for all comparisons), as compared to the NCsiRNA and DEPC-injected control (Fig. 2A).

Analogously, the relative *Cyp6g2* transcript levels were decreased by 1.5-, 1.6-, and 2-fold at 24 h, 48 h, and 72 h after injection of mimics, respectively (*F* = 16.1, df = 8, *p* < 0.05 for all comparisons), as compared to the NCsiRNA and DEPC-injected control (Fig. 2B). The *Cyp6g1* transcripts showed the lowest relative level at 72 h post *miR-310s* injection as compared to 24 h and 48 h post injection (*p* < 0.05). However, there was no significant difference in the expression of *Cyp6g2* transcripts across three time points in the mimic-injected group (*p* = 0.879–0.998).

### Validation of *miR-31*0s-mediated regulation of *Cyp6g1* and *Cyp6g2* transcripts in vitro

**Figure 3:**
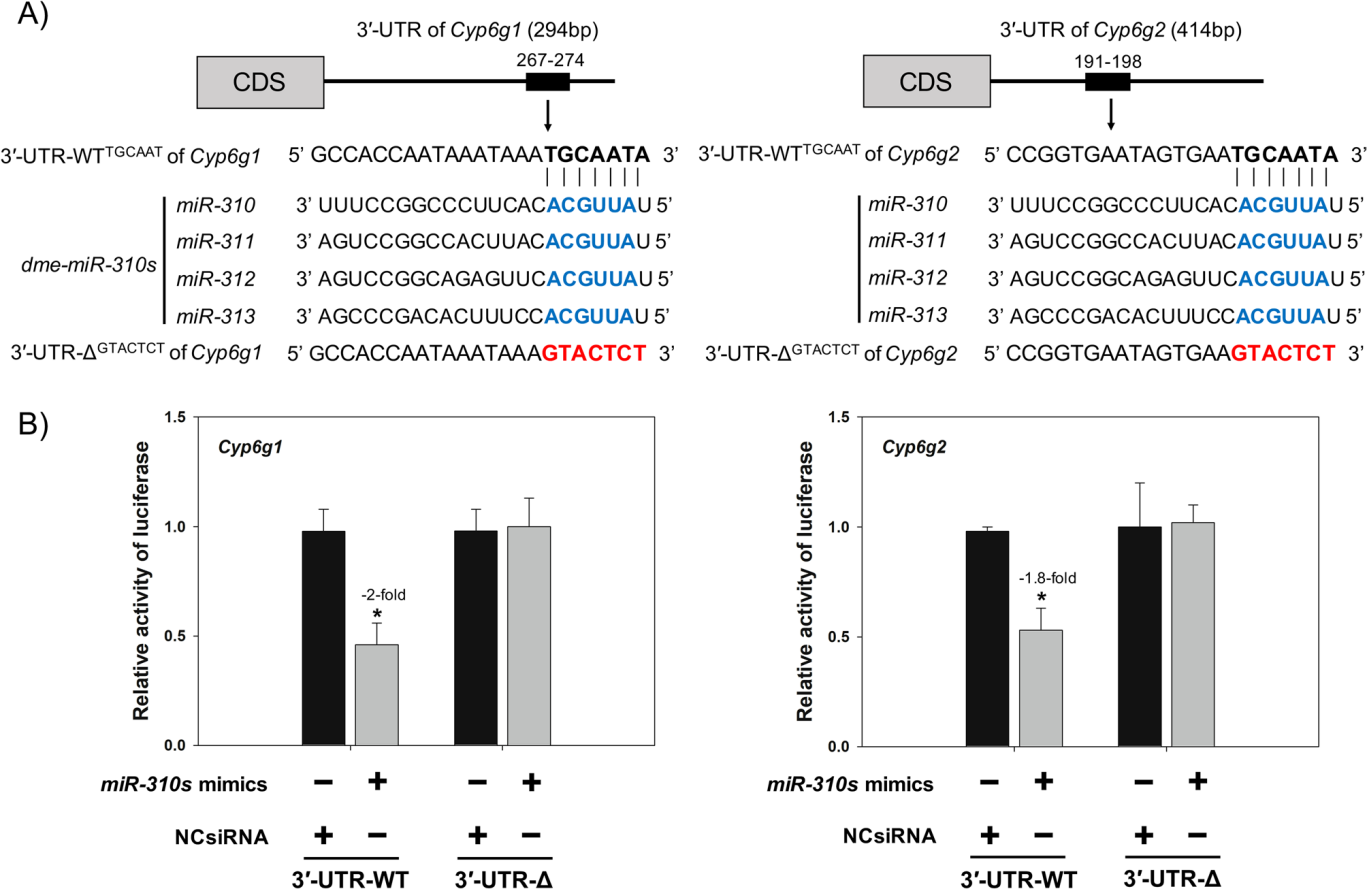
Validation of *miR-310s* -mediated regulation of *Cyp6g1* and *Cyp6g2* transcripts in vitro. (**A**) Predicted target binding site (3′-UTR-WT^TGCAAT^) and mutated target binding site (3′-UTR-Δ^GTACTCT^) of *miR-310s* within the 3′-UTR of the putative target *Cyp6g1* and *Cyp6g2* from *91-R* strain. (**B**) Relative luciferase activity in S2 cells co-transfected with *miR-310s* or negative control mimics and the wide- or mutant-type luciferase reporter vectors (3′-UTR-WT^TGCAAT^ and 3′-UTR-Δ^GTACTCT^). An asterisk (*) indicate a difference across the treatment groups at *p* < 0.05.

The *miR-310s* cluster shares an identical seed sequence among *miR-310*, *miR-311*, *miR-312*, and *miR-313* within the 3ʹ-UTRs of both *Cyp6g1* and *Cyp6g2* (Fig. 3A). Specifically, the seed sequences for *miR-310s*, ACGUUA, were located at positions of 267 to 274 and 191 to 198 of the 3ʹ-UTR for *Cyp6g1* and *Cyp6g2*, respectively. The putative transcript target sites showed 100% complementarity to the seed regions, ACGUUA, for all endogenous and mimic *miR-310s* sequences. Assays from S2 cells co-transfected with dual reporter psiCHECK-2-3ʹ-UTR-WT^TGCAAT^ constructs + *miR-310s* mimics resulted in a decrease in luciferase activity; specifically, assays using wild-type 3ʹ-UTRs (3ʹ-UTR-WT^TGCAAT^) of *Cyp6g1* and *Cyp6g2*, respectively, showed an approximate reduction of 2-fold (*F* = 8.2, df = 1, *p* < 0.05) and 1.8-fold (*F* = 125.5, df = 1, *p* < 0.05) as compared to the NCsiRNA treatment (Fig. 3B). However, no significant change in luciferase activity was detected for experiments that were co-transfected with the *miR-310s* mimics along with the constructs containing the mutant 3ʹ-UTR-Δ^GTACTCT^ seed regions from *Cyp6g1* (*F* = 0.01, df = 1, *p* = 0.918) or *Cyp6g2* (*F* = 0.167, df = 1, *p* = 0.704) as compared with the NCsiRNA treatment (Fig. 3B).

### Impact of *miR-31*0s modulation on DDT-induced mortality

To validate the putative involvement of increased levels of *miR-310s* mimics in DDT resistance in the *91-R* strain, mortality bioassays were performed at 24 h post-injection of adults with *miR-310s* mimics. Probit analyses demonstrated that the *miR-310s* mimics injected flies exhibited a LT_50_ value of 17.0 h (χ^2^ = 81.1, df = 2, *p* < 0.01), which was a shorter time span as compared to the NCsiRNA treatment (28.5 h; χ^2^ = 64.9, df = 2, *p* < 0.01) and the DEPC-water control (29.1 h; χ^2^ = 59.1, df = 2, *p* < 0.01) (Table 1). However, evidenced by the overlapping 95% CL, the NCsiRNA injected flies did not exhibit any statistically significant difference for their LT_50_ value as compared to the DEPC-water control treatment.

**Table 1:** Evaluation of DDT sensitivity in *Drosophila 91-R* strain after *miR-310s* mimics injection using a topical exposure to DDT (0.5 µg/fly).

Treatment	LT_50_^a^ (hour)	95% C.L.^b^	χ^2^ (df)	Slope ± SE	*P* value
*miR-310s* mimics	17.0	12.0–21.3	81.1 (2)	2.2 ± 0.41	< 0.01
NCsiRNA	28.6	22.1–37.3	65 (2)	2.0 ± 0.44	< 0.01
DEPC-water control	29.1	21.6–38.8	59.1 (2)	1.98 ± 0.5	< 0.01

^a^Lethal Time 50 (hour) that killed 50% of the flies.

^b^95% Confidence limit.

**Figure 4:**
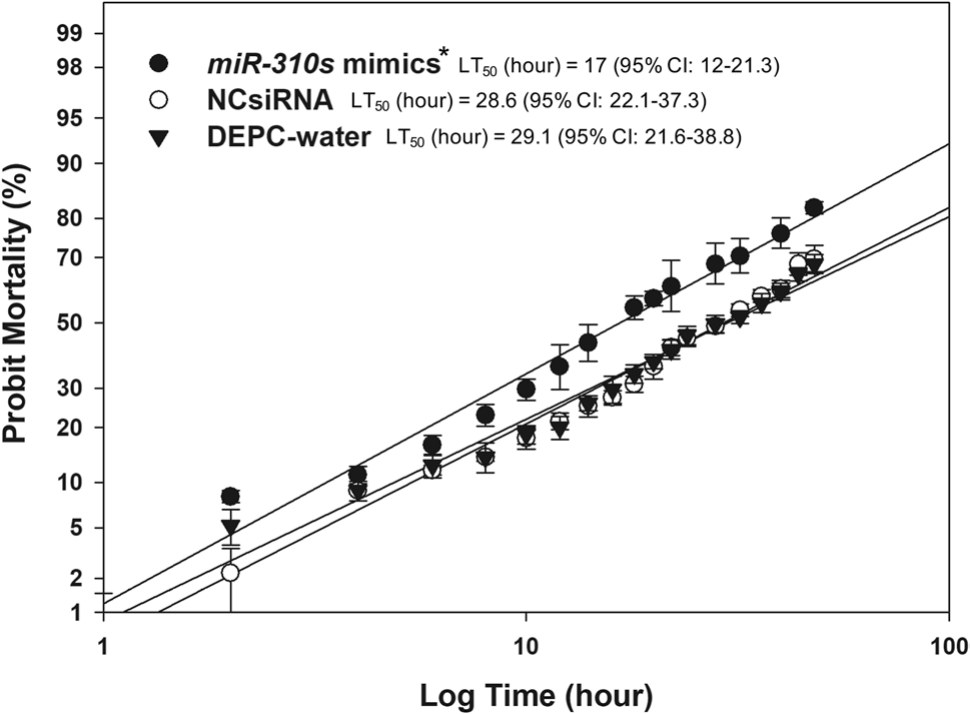
Comparative DDT (0.5 μg/fly) induced mortality of microinjected *91-R* female flies with *miR-310s* mimics, negative control siRNA mimic (NCsiRNA), and DEPC-water. There was no difference between the DEPC-water and NCsiRNA microinjection groups (*p* = 0.263). The Fisher *F*-test was performed to verify if the regression lines were equal to each other. An asterisk (*) indicates a difference across the treatment groups at *p* < 0.05. 95% CI: 95% Confidence limit.

Using the Fisher *F*-test, we observed that the regression lines between the *miR-310s* mimics injection group and NCsiRNA group were significantly different (*F* = 14.5, df = 3, *p* < 0.01; Fig. 4). Analogously, the estimated regression line for the *miR-310s* mimics injection treatment were different than the DEPC-water control treatment (*F* = 12.1, df = 3, *p* < 0.0001; Fig. 4). In contrast, results of the *F*-test predicted the relative equality between regression lines between the NCsiRNA and DEPC-water control groups (*F* = 1.4, df = 3, *p* = 0.263; Fig. 4). Additionally, the acetone-only negative control showed no fly mortality (data not shown).

### Polymorphisms in TF-biding motif putatively associated with expression level of *miR-31*0s

**Figure 5:**
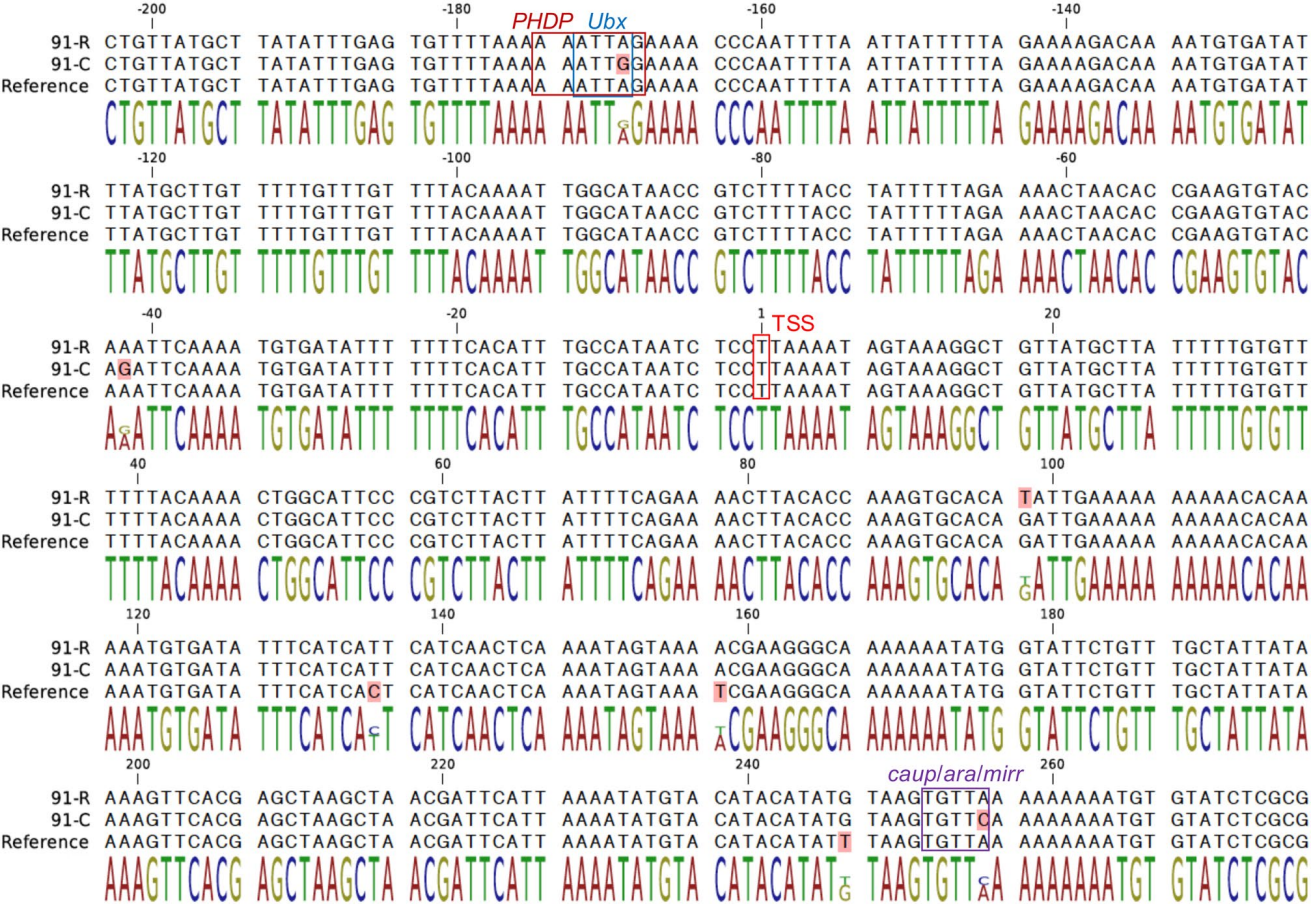
The position of putative transcription factor binding sites within 200 bp up-stream and 280 bp down-stream of the predicted *miR-310s* cluster transcription start site (TSS). The transcription factor binding sites are boxed. The polymorphisms among three strains are shaded in red.

The predicted transcriptional start site (TSS) of the *miR-310s* cluster was located 268 bp upstream of the first miRNA, *miR-313*, but no other TSSs were observed within the cluster. Using the JASPAR database, a total of 216 different putative TF-binding motifs were predicted in the *cis*-regulatory region of the *miR-310s* cluster with relative profile score threshold 95% (Supplemental Table S1). A comparison of nucleotide sequences from this region, derived from short read alignments between DDT-resistant *91-R* and DDT-susceptible *91-C*, predicted that two mutation sites were within three putative TF-binding motifs upstream of the *miR-310s* cluster. Specifically, predicted binding motifs for *PHPD* and *Ubx* contain a single nucleotide polymorphism (A for *91-R* and G for *91-C*), respectively (Fig. 5). Additionally, *caup*/*ara*/*mirr* binding motif contains a change in single nucleotide from A for *91-R* to C for *91-C* (Fig. 5).

## Discussion

Arthropods continue to damage agricultural commodities and vector diseases threatening human welfare due to ongoing challenges in pest control that arise from instances where selection has caused high levels of resistance following repeated exposures to insecticidal agents. The involvement of miRNAs in the regulation of metabolic resistance to insecticides has been suggested via comparative and correlative studies for pyrethroids^29^ and ryanoids^30^, but strong evidence for miRNAs as causative factors among these resistant phenotypes is arguably lacking. The present study demonstrated that members for the *miR-310s* cluster interact with target binding sites within 3ʹ-UTR sequences of cytochrome P450, thereby regulating transcript levels in vivo. Specifically, we showed here that transcript from two cytochrome P450 genes, *Cyp6g1* and *Cyp6g2*, are up-regulated in the DDT-resistant strain *91-R* as compared to the -susceptible strains *91-C* and *Canton-S*, reconfirming our previously published results^12,31^.

Previous studies provide supporting evidence that high to moderate level DDT resistance is polygenic in *Drosophila*, with multiple resistance genes, including P450s, associated with the DDT resistance phenotype^8–10^. The *Cyp6* subfamily has been associated with DDT resistance and cross-resistance to neonicotinoid insecticides^32,33^. For example, statistically significant differences in expression were documented for *Cyp6a2*, *Cyp6a8*, *Cyp6g1*, *Cyp6g2*, and *Cyp6w1* in DDT-resistant *91-R* and *Wisconsin* strains as compared to DDT- susceptible *Canton-S* and *91-C* strains; relatedly, previous evidence of significant changes in expression of *Cyp6* subfamily is functionally involved in DDT resistance in *Drosophila*^12,19,34^. Moreover, overexpression of the CYP6W1-Ala370 allele in transgenic *Drosophila* was sufficient to confer low levels of DDT tolerance relative to CYP6W1-Val370 and CYP6W1-Gly370^16^. Many of these genes, however, were not found to be differentially expressed in field-derived resistance strains^35^. The one exception was found in prior experiments that genetically mapped the positioned locus of major effect on DDT resistance within a genome region encoding *Cyp6g1*^18,33^. These authors also documented that *Cyp6g1* was up-regulated in resistant adult *Drosophila* flies^18^. Furthermore, expression of the *Cyp6g1* enzyme in a heterologous system was capable of carrying out a dichlorination step in the metabolism of DDT^36^, indicating a likely role of the translated protein in the cellular detoxification mechanism.

In keeping with these previous studies, our results support the hypothesis that the *miR-310s* cluster mediate the regulation of *Cyp6g1* and *Cyp6g2* transcript levels, as well as part of the DDT resistance phenotype in the *91-R* strain. These results advance research based on our previous study that reported the *miR-310s* are down-regulated in the DDT-resistant *91-R* strain as compared to its susceptible counterpart *91-C*^21^*.* Equally, the expression of the *miR-310s* was inversely correlated with the up-regulation of a number of detoxification genes (P450s, GSTs, and esterases) that also had computationally-predicted *miR-310s* target binding sites in their 3ʹ-UTRs^21^. Specifically, validation experiments here showed a reduction in *Cyp6g1* and *Cyp6g2* transcript levels following injection of *miR-310s* mimics into adult *91-R* females (Figs. 1, 2), hypothetically via target-specific degradation via the RISC pathway^37^. Although a range of other target genes could be influenced by *miR-310s* mimic injection, the resulting reduction in *Cyp6g1* and *Cyp6g2* transcript levels and concomitant more rapid mortality (median lethal time; LT_50_) within the *miR-310s* injected group as compared to the control-injected group from strain *91-R* (Fig. 4) suggests changes in one or both *Cyp6g1* and *Cyp6g2* transcripts contributes to a portion of the observed changes in DDT susceptibility.

However, we cannot firmly establish any direct involvement of *miR-310*-mediated regulation of *Cyp6g2* on the DDT resistance phenotype in *91-R*. Specifically, although the role of *Cyp6g1* in DDT resistance is established, neither is any direct role of *Cyp6g2* in DDT resistance yet reported from *Drosophila,* nor is there direct functional evidence for enzymatic products of *Cyp6g2* in DDT detoxification. However, a number of previous studies suggested that the overexpression of *Cyp6g2* gene was associated with resistance to several insecticides such as imidacloprid, ivermectin, and diazinon in *Drosophila*^35,38,39^. Moreover, the significant overexpression of *Cyp6g2* was associated with the DDT-resistant *91-R* compared to susceptible strains *Canton-S* and *91-C*^12,19,31^. Additionally, the current study provides empirical evidence demonstrating a direct impact of *miR-310s* on corresponding transcript levels of *Cyp6g1* and *Cyp6g2* and DDT resistance in *91-R*. Regardless, disentangling the independent effects of *Cyp6g1* and *Cyp6g2* on DDT resistance remains unresolved*.* Expression of *Cyp6g1* and *Cyp6g2* is not only highly correlated, which was suggested to be a consequence of physically linked allelic variants^38,40^ but also shown to results from impacts of the *miR-310* cluster. Therefore, it remains plausible that *Cyp6g2* associations with DDT may be a consequence of genomic proximity to and recent co-ancestry with *Cyp6g1. The* independent role of *Cyp6g2* in DDT resistance remains to be investigated through future functional experiments.

Evidence from our microinjection experiments suggested a direct involvement of *miR-310s* in regulation of *Cyp6g1* and *Cyp6g2*, and concomitant mediation of at least a portion of the DDT resistance phenotype in female *91-R Drosophila*. Previously, Chung, et al.^41^ demonstrated that the up-regulation of *Cyp6g1* is associated with the insertion of an *Accord* retrotransposon within the upstream region of the *Cyp6g1* in a resistant field strain. Moreover, Schmidt et al. also found that *Cyp6g1* increased expression was influenced by copy number variation^42^ and that these structural variants are an outstanding feature in *Cyp6g1* associated with DDT resistance^43^. Additionally, a functional *Nrf2*/*Maf* (NF-E2-related factor 2/Muscle aponeurosis fibromatosis) transcription factor can enhance the constitutive up-regulation of *Cyp6a2* and *Cyp6a8* transcription, which also was associated with DDT resistance in *Drosophila*^44,45^. Furthermore, a nucleotide mutation in the estrogen-related receptor (*ERR*) gene led to over-expression of *Cyp12d1* and *Cyp6g2* in *Drosophila*^46^. These aforementioned lines of evidence demonstrate that the regulation of transcript levels for P450s, as exemplified with *Cyp6g1* and *Cyp6g2*, may involve multiple regulatory factors; moreover, the genetic background of the flies may influence which one (or several) of these factors cause constitutive over-transcription. Current evidence supports the hypothesis that *miR-310s* may play a role in post-transcriptional longevity of *Cyp6g1* and *Cyp6g2* transcripts in DDT-resistant *91-R* strain and the corresponding higher turnover of these transcripts in susceptible counterparts *91-C* and *Canton-S* strains.

Despite documenting that *miR-310s* are likely causative of a portion of the DDT resistance phenotype in *91-R*, demonstration of a putative *miR-310s* seed sequence dependence of transcript quantities in vitro were lacking. In order to address this shortfall, a luciferase assay verified that an intact *miR-310s* target binding sites in the 3ʹ-UTR of both *Cyp6g1* and *Cyp6g2* is necessary and sufficient for the degradation of corresponding transcript levels within S2 cells. Specifically, we revealed that the relative luciferase activity of wild type 3ʹ-UTR (WT^TGCAAT^) for both *Cyp6g1* and *Cyp6g2* was reduced when co-expressed with *miR-310s* mimics, but this effect was not seen for the mutant version, Δ^GTACTCT^ (Fig. 3). This suggested that the functional target binding sites in 3 ʹ -UTRs of *Cyp6g1* and *Cyp6g2* mRNAs likely facilitate complementary base pairing with *miR-310s* and, in turn, may impact stability or degradation of *Cyp6g1* and *Cyp6g2* mRNAs.

The role of miRNAs in regulating P450 gene expression has also been investigated in several other insect species. Prior work supports the hypothesis that *miR-2b-3p* regulates the expression of two P450s, *Cyp9f2* and *Cyp307a1*, putatively involved in deltamethrin resistance in *Plutella xylostella*^29^. *Let-7* and *miR-100* have been proposed to modulate the post-transcriptional regulation of *Cyp6cy3* gene to alter the tolerance of *Myzus persicae nicotianae* to nicotine^47^. The upregulation of *miR-285* is thought to increase resistance to deltamethrin in *Culex pipiens pallens* by binding to 3′-UTR region of *Cyp6n23* resulting in changing in expression of *Cyp6n23*^48^, but direct evidence was not provided. In this study, we provide functional evidence demonstrating a likely role of *miR-310s* species in the binding and subsequent stability of putatively targeted cytochrome P450s and is the first known instance of making such a connection for a gene shown to have a role in mediating the expression of a DDT resistance trait.

The molecular genetic or biochemical basis of resistance has been resolved in cases where traits are monogenic or share a conserved mechanism across genera^49,50^. However, phenotypes showing a high level of resistance based on polygenic mechanisms have been more difficult to elucidate. The laboratory DDT-selected *Drosophila* strain *91-R* has served as a model system to understand the molecular mechanisms underlying polygenic pesticide resistance^14,31,51,52^. Thus, the increased levels of *Cyp6g1* and *Cyp6g2* transcripts, and assumed relative increases in corresponding protein levels, mediated by reduced degradation by down-regulated *miR-310s* likely contributes partially to the DDT resistance phenotype in *91-R*. This conclusion is based on the phenotypic response of *91-R* following injection of *miR-310s* mimics (Fig. 4), as well as evidence that other genes are functionally involved, such as the phase III detoxification ATP binding cassette transporter, *Mdr49*^13^. Furthermore, RNAi mediated knockdown of several phase I (*Cyp6g1*, *Cyp12d1*, and *Cyp4g1*), phase III (*Mdr49*, *Mdr50*, *Mdr65*, and *Mrp1*), and other cuticular (*Lcp1*) genes documented their capacity to contribute to DDT resistance in *91-R*^8^. Moreover, DDT resistance in *91-R* is dependent upon genetic factors located within 13 major and 3 minor genome regions affected by selective sweeps^14^, as well as changing in multiple pathways that impact neuronal function and cell stress response^31^. Our DDT bioassay results support the hypothesis that *miR-310s* -mediated expression of *Cyp6g1* and *Cyp6g2* contributes to the polygenic DDT resistant phenotype in *91-R*.

The possibility that the reduced expression of *miR-310s* in *91-R* may impact a range of other genes cannot be ruled out. Computational predictions suggest putatively regulated transcripts encode a broad range of proteins, including a subset with functional annotation suggesting roles in xenobiotic detoxification^21^. Thus, the role of *miR-310s* in the DDT resistance phenotype may be more complex, and a future genome-wide transcriptome analysis of *miR-310s* mimics injected *Drosophila* samples could potentially provide a precise and unbiased measure of impact on target transcripts.

The impact of genetic variants on miRNA expression and function still remain unclear. A number of studies analyzing genome-wide nucleotide and miRNA expression variation identified putative polymorphisms significantly associated with the regulation of miRNA expression^53,54^. In this study, nucleotide mutations were identified in the *cis*-regulatory region of putative TF-binding motifs from the *miR-310s* cluster. However, the impact of these alterations on transcript expression levels of the *miR-310s* cluster and their contribution to DDT resistance phenotypes in *Drosophila* remain to be investigated.

Regardless, despite the other unknown molecular function(s) of *miR-310s*, the current study establishes a role of *miR-310s* in modulating cellular levels of *Cyp6g1* and *Cyp6g2* transcripts. Due to the co-repression of these two transcripts via *miR-310* mimics, their independent effects on subsequent DDT resistance levels cannot be disentangled, such that the novel possibility of *Cyp6g2* involvement in the resistance phenotype of *91-R* could not be dissected using our methodology. Furthermore, since there is a paucity of in vivo and in vitro functional studies that aim to uncover protein function or the complex regulatory networks interconnecting miRNA and target mRNAs, the identification of novel genes and pathways (and both their direct and indirect consequences) are needed. Such studies will undoubtedly assist in the elucidation of relationships for miRNA-based gene regulation and DDT resistance in *Drosophila* and potentially shed light on analogous mechanisms across arthropods.

## Materials and methods

### *Drosophila* strains

The DDT-susceptible *Canton-S*, the low-level DDT-resistant *91-C*, and the highly DDT-resistant *91-R* strains of *Drosophila* have been maintained for almost two decades in the Pittendrigh laboratory (Michigan State University, East Lansing, MI, USA). The *91-R* strain has been continually reared as described previously^31^, with selection maintained by growing flies in the presence of 150 mg DDT impregnated paper filter disks while *Canton-S* and *91-C* were maintained without any exposure to DDT. The *91-R* strain has been shown to be ~ 1,500-fold and ~ 107-fold resistant to DDT compared to the susceptible *Canton-S* and *91-C* strains through the use of contact and topical bioassays^31,55^. In order to compare the constitutive expression of miRNAs and cytochrome P450 genes in subsequent analyses (see below), all flies were not exposed to DDT within that generation.

### Microinjection of female adult *Drosophila* and RNA isolation

A set of *mirVana* mimics were synthesized for *miR-310*, *miR-311*, *miR-312*, and *miR-313* by Invitrogen (Ambion, Life Technologies) at a concentration of 100 µM. Members of the *miR-310s* cluster are positioned less than 1 kb apart and show 100% homology between independent seed sequences. Clustered miRNAs are often co-expressed^56,57^ and co-regulate functionally related genes^58,59^, therefore were treated as a single functional unit of study within our experiments. One-day-old *91-R* female flies were anesthetized on ice, and 69 nl of combined all four mimics, *miR-310s*, (final concentration 25 µM), were injected into the side of the thorax of individual *Drosophila* adults using 2-in. needles using a Drummond’s Nanoject II microinjector (Drummond Scientific Company, USA). AllStars Negative Control siRNA (NCsiRNA; Qiagen, Valencia, CA) were similarly injected into female flies with the same volume and concentration as the mimics of *miR-310s* treatments. Corresponding negative controls consisting of the diethylpyrocarbonate (DEPC)-treated water were analogously injected. After they were microinjected, the flies were immediately placed into small plastic tubes and allowed to recover at 25 °C and 50%-70% humidity with 16/8 h day-night light cycle with commercially available medium (Jazz-Mix *Drosophila* Food, Fischer Scientific). Each experiment was performed in triplicate with independent microinjections. Total RNA was extracted from a pool of fifteen flies from each replicate of mimic, NC-siRNA, and DEPC-water injected groups at 24 h, 48 h, and 72 h post-microinjections using the Qiagen miRNeasy Mini Kit according to the manufacturer’s instructions (Qiagen). Each sample was treated with DNase I (Zymo Research, Orange, CA) to remove contaminating genomic DNA prior to cDNA synthesis.

### Gene expression by Reverse Transcriptase-quantitative PCR (RT-qPCR)

First-strand cDNA synthesis was performed using the miScript II RT kit (Qiagen). Each synthesized cDNA sample was then used as a template for RT-qPCR reactions using the miScript SYBR Green PCR Kit (Qiagen) according to the manufacturer’s instructions with miRNA-specific forward primers (Table 2). The same cDNA template was analogously used in RT-qPCR reactions primed by forward and reverse primers for corresponding putative targeted P450 transcripts (Table 2) using the Power SYBR Green PCR Master Mix according to the manufacturer’s instructions (Applied Biosystems Inc., Foster City, CA). All RT-qPCR amplification reactions were performed on a StepOnePlus Real-Time PCR system (Applied Biosystems Inc.), with three technical replicates across all biological replicates. Normalization of the relative expression levels of each *miR-310s* and target cytochrome P450s was made with respect to the reference genes, *5S rRNA* and *rp49*, respectively. Normalized miRNA and target transcript expression levels were calculated using the 2^−∆∆C(t)^ method^60^. Statistical analysis was performed using a one-way analysis of variance (ANOVA) by Tukey’s multiple sample comparisons using XLSTAT software (Addinsoft, NY, USA), and a significance threshold set at *p* < 0.05.

**Table 2:** Sequences of the primers used in this study.

miRNA or gene	Forward primers	Reverse primers	Remarks
*5S rRNA*	CGACCATACCACGCTGAATA	Universal primer (supplied from miScript SYBR Green PCR Kit)	RT-qPCR
*miR-310-3p*	UAUUGCACACUUCCCGGCCUUU	Universal primer (supplied from miScript SYBR Green PCR Kit)	
*miR-311-3p*	UAUUGCACAUUCACCGGCCUGA	Universal primer (supplied from miScript SYBR Green PCR Kit)	
*miR-312-3p*	UAUUGCACUUGAGACGGCCUGA	Universal primer (supplied from miScript SYBR Green PCR Kit)	
*miR-313-3p*	UAUUGCACUUUUCACAGCCCGA	Universal primer (supplied from miScript SYBR Green PCR Kit)	
*Rp49*	CGGATCGATATGCTAAGCTGT	GCGCTTGTTCGATCCGTA	
*Cyp6g1*	GAATTCGCACCAAGCTGACT	TCCCAGAGTTCTTCTCTCCA	
*Cyp6g2*	ATGTAGGTGTAGGGCGTGT	CAAGGGCATGCCCGTTTATA	
*Cyp6g1* 3′UTR	*XhoI*: tccgctcgagATTTGAATCGCATGAACTGTG	*NotI*: agaatgcggccgcATAATCGTAAAGATAGCATTT	Vector construction
*Cyp6g2* 3′UTR	*XhoI*: tccgctcgagAGCTGGTGTCGCATCTTAAA	*NotI*: agaatgcggccgcTGAGCAGCTAGCAGCTACTC	

### Construction of luciferase reporter vectors and luciferase assay

The psiCHECK-2 dual fluorescent reporter system (Promega, Madison, WI, USA) was used to determine any interaction of *miR-310s* with putative target binding sites in *Cyp6g1* and *Cyp6g2*. The psiCHECK-2 system incorporates *Renilla* luciferase as a reporter gene and firefly luciferase as a control to normalize for transfection efficiency and cell number in the reporter gene assay. Constructs in the current study were designed to contain either the 3ʹ-UTR of wild-type *Cyp6g1* or *Cyp6g2*, or a mutant version of each, cloned upstream of the *Renilla* luciferase reporter gene. For this aforementioned cloning, a partial region of the wild-type 3ʹ-UTR including target binding sites of *miR-310s* cluster for *Cyp6g1* and *Cyp6g2* were PCR amplified from *91-R* isolated gDNA using the primers incorporating 5ʹ extensions with *XhoI* and *NotI* restriction endonuclease recognition sites (Table 2). All PCR reactions were carried out using Phusion High-Fidelity DNA Polymerase (New England Biolabs, Ipswich, MA) with *91-R* genomic DNA as the template. Each PCR product was purified using the QIAquick PCR Purification Kit (Qiagen) and then ligated into the pGEM-T Easy Vector Systems (Promega). The subsequent transformed clones were purified with a QIAprep Miniprep kit (Qiagen). After sequence analysis, the purified plasmid products were digested using the *XhoI* and *NotI* enzymes (New England Biolabs) and then cloned into the downstream of *Renilla* luciferase reporter gene in psiCHECK-2 plasmid vector (Promega). The putative *miR-310s* binding site within *Cyp6g1* and *Cyp6g2* 3′-UTRs, TGCAAT, was altered to GTACTCT (Δ^GTACTCT^) using the Q5 Site‐Directed Mutagenesis Kit (NEB). Mutant *Cyp6g1* and *Cyp6g2* 3ʹ-UTR *Renilla* luciferase reporter gene psiCHECK-2 constructs were generated as described above. All the constructs were verified by Sanger sequencing in both directions.

*Drosophila* Schneider 2 (S2) Cells from the *Drosophila* Genomics Resource Center (DGRC, Bloomington, IL) were obtained, and cultured in Schneider’s *Drosophila* medium (GIBCO, Rockville, MD) containing 10% fetal bovine serum (FBS; VWR, Radnor, PA), 50 units/ml penicillin, and 50 μg/ml streptomycin at 27 °C. The S2 cells were seeded at a density of 4 X 10^5^ cells per well in 6-well plates and were cultured for 24 h to 80% confluence. The cells were divided into four groups for the following four treatments: (1) psiCHECK-2_3ʹ-UTR-WT^TGCAAT^ + *miR-310s* mimics; (2) psiCHECK-2_3ʹ-UTR-WT^TGCAAT^ + NCsiRNA (control); (3) psiCHECK-2_3ʹ-UTR- Δ^GTACTCT^ + *miR-310s* mimics; and, (4) psiCHECK-2_3ʹ-UTR- Δ^GTACTCT^ + NCsiRNA (control). S2 cells were co-transfected with 0.5 μg of each reporter construct and 100 nM miRNA of the mimics or NCsiRNA per well using the FuGENE HD Transfection Reagent (Promega). The firefly and *Renilla* luciferase activities were assayed with the Dual-Luciferase Assay System (Promega) after 48 h according to the manufacturer’s protocols. The *Renilla* luciferase activity was normalized by firefly luciferase activity. All experiments were repeated at least three times. Significant differences were determined based on ANOVA followed by Tukey's HSD multiple comparison test (*p* < 0.05).

### DDT sensitivity bioassay

One-day-old female *91-R* (90 flies per each injection) were injected separately with *miR-310s* mimics, NCsiRNA, and DEPC-water using microinjection methods described above and allowed one day for recovery. A 2.5 mg ml^−1^ solution of DDT dissolved in acetone was prepared, and then 0.2 µl (0.5 µg DDT per fly) was topically applied to the pronotum of female flies using a 50 µl glass micropipette (Hamilton 705SNR, Reno, NV) fitted in a repeating dispenser (Hamilton PB-600). Treated flies were transferred to 20 ml glass vials and capped with cotton plugs moistened with 5% sucrose solution in distilled water. Acetone-only treated flies were used as a control. The effect of DDT toxicity on flies was assessed to determine if the flies were unable to fly and crawl over the inner surface of the glass vial. Flies that remained immobile on the bottom of vial with slow leg twitching were considered dead. Median lethal time (LT_50_) was estimated for each treatment group based on the regression of Log_10_ time versus PROBIT percent mortality using the statistical software XLSTAT (Addinsoft). Each bioassay was repeated three times with independent microinjections. The three logistic regression curves were examined by F-test to determine whether any differences in the resulting mortality curves from differently treated fly groups were statistically significant (*p* < 0.01).

### Analysis of putative transcription factor (TF)-binding-motifs of *miR-31*0s

The putative promoter regions were analysed for the potential *cis*-acting elements and motifs of *miR-310s* cluster which could potentially account for expression changes in strain *91-R* as compared to *91-C*. The transcription start site (TSS) of the *miR-310s* cluster was predicted for the *Drosophila* genome release 6 by *McPromoter*^61^. A 480 bp region containing 200 bp up-stream and 280 bp down-stream of the predicted *miR-310s* cluster transcription start site (TSS) was excised from the *Drosophila* reference genome assembly release 6, in which putative regulatory element motifs were predicted using the JASPAR database release 7 online query tool^62^ with an applied relative profile score threshold of 95%. Default parameters of the ‘Map Reads to Reference’ tool of the CLC genomic workbench v.12.0 (Qiagen) were used to map Illumina short genomic sequence reads from *91-C* and *91-R* [SRA accession: SRX516723 and SRX516724]^14^ to the excised upstream regulatory regions of *miR-310s* cluster. The consensus nucleotide sequence of *miR-310s* cluster from each *91-R* and *91-C* were aligned with the corresponding region of the *Drosophila* reference sequence release 6 using the Alignments and Tree tool of the CLC Genomic Workbench (Qiagen), and polymorphism within putative *cis*-regulatory TF binding sites predicted by JASPAR were identified manually.

## Supplementary information

Supplementary Information.
