# Supplementary material for: Post-transcriptional modulation of cytochrome P450s, Cyp6g1 and Cyp6g2, by miR-310s cluster is associated with DDT-resistant Drosophila melanogaster strain 91-R
Source: Sci Rep. 2020 Sep 1;10:14394. doi: 10.1038/s41598-020-71250-0 (PMC7463240; doi:10.1038/s41598-020-71250-0)
Supplement: Supplementary file 1 — Supplementary Information. [file 41598_2020_71250_MOESM1_ESM.pdf]

**Supplementary Information**

# Post-transcriptional modulation of cytochrome P450s, *Cyp6g1* and *Cyp6g2*, by *miR-310s* cluster is associated with DDT-resistant *Drosophila melanogaster* strain *91-R*

Keon Mook Seong[1*], Brad S. Coates[2], Barry R. Pittendrigh[3]

[1] Department of Applied Biology, College of Ecology and Environment, Kyungpook National University, Sangju, Korea

[2] USDA-ARS, Corn Insects & Crop Genetics Research Unit, Ames, IA, USA

[3] Department of Entomology, Michigan State University, East Lansing, MI, USA

**Supplementary Figure S1.** Estimates of mean transcription level for *Cyp6g1* and *Cyp6g2* genes in female DDT-resistant *91-R* and -susceptible *91-C* relative to the control DDT-susceptible strain *Canton-S*. The vertical bars indicate standard error of the mean (SEM). Different letters on the bars indicate that the means are significantly different among three strains ($p < 0.05$).

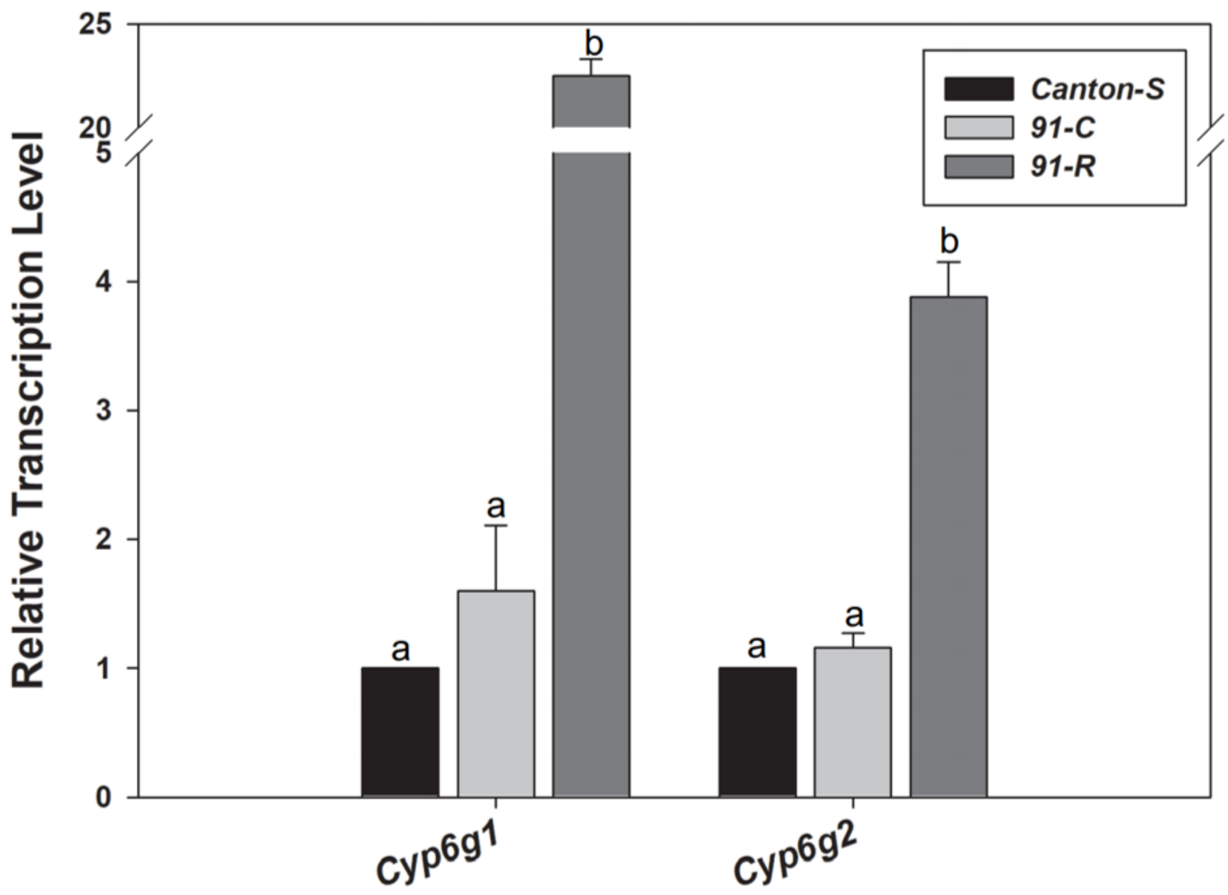

**Supplementary Table S1.** Predicted transcription factor binding sites identified within *miR-310s* cluster.

| Matrix ID | Name | Score | Relative score | Sequence ID | Start | End | Strand | Predicted sequence |
|---|---|---|---|---|---|---|---|---|
| MA0231.1 | lbe | 8.47166 | 1 | 310s_upstream | 27 | 32 | + | TAACTA |
| MA0094.1 | Ubx | 6.91669 | 1 | 310s_upstream | 31 | 34 | + | TAAT |
| MA0094.1 | Ubx | 6.91669 | 1 | 310s_upstream | 34 | 37 | + | TAAT |
| MA0182.1 | CG4328-RA | 9.08537 | 0.996111 | 310s_upstream | 31 | 37 | - | ATTATTA |
| MA0210.1 | ara | 6.45422 | 0.950245 | 310s_upstream | 38 | 42 | + | ATACA |
| MA0168.1 | B-H1 | 9.90717 | 0.97745 | 310s_upstream | 41 | 47 | - | TTAAATG |
| MA0170.1 | C15 | 8.82136 | 0.966032 | 310s_upstream | 41 | 47 | - | TTAAATG |
| MA0233.1 | mirr | 7.77238 | 1 | 310s_upstream | 51 | 55 | - | AAACA |
| MA0210.1 | ara | 7.42747 | 0.99577 | 310s_upstream | 51 | 55 | - | AAACA |
| MA0185.1 | Deaf1 | 6.96107 | 0.958761 | 310s_upstream | 65 | 70 | + | TTCGTT |
| MA0094.1 | Ubx | 6.91669 | 1 | 310s_upstream | 72 | 75 | + | TAAT |
| MA0457.1 | PHDP | 9.14108 | 0.966907 | 310s_upstream | 71 | 77 | + | TTAATTT |
| MA0187.1 | Dll | 8.9745 | 0.958924 | 310s_upstream | 72 | 78 | + | TAATTTC |
| MA0204.1 | Six4 | 8.60921 | 0.959545 | 310s_upstream | 84 | 89 | + | TGAGAC |
| MA0094.1 | Ubx | 6.91669 | 1 | 310s_upstream | 97 | 100 | - | TAAT |
| MA0232.1 | lbl | 8.49402 | 0.97079 | 310s_upstream | 95 | 100 | - | TAATGA |
| MA0215.1 | btn | 9.67421 | 0.956897 | 310s_upstream | 95 | 101 | - | ATAATGA |
| MA0197.1 | nub | 9.56968 | 0.956695 | 310s_upstream | 95 | 102 | - | TATAATGA |
| MA0210.1 | ara | 6.45422 | 0.950245 | 310s_upstream | 100 | 104 | + | ATACA |
| MA0210.1 | ara | 6.45422 | 0.950245 | 310s_upstream | 169 | 173 | - | ATACA |
| MA0094.1 | Ubx | 6.91669 | 1 | 310s_upstream | 172 | 175 | - | TAAT |
| MA0182.1 | CG4328-RA | 9.08537 | 0.996111 | 310s_upstream | 172 | 178 | + | ATTATTG |
| MA0233.1 | mirr | 7.77238 | 1 | 310s_upstream | 206 | 210 | + | AAACA |
| MA0210.1 | ara | 7.42747 | 0.99577 | 310s_upstream | 206 | 210 | + | AAACA |
| MA0254.1 | vvl | 8.27074 | 1 | 310s_upstream | 218 | 223 | - | TATGCA |
| MA0235.1 | onecut | 10.8156 | 1 | 310s_upstream | 252 | 258 | + | TTGATTT |
| MA0227.1 | hth | 9.78892 | 1 | 310s_upstream | 278 | 283 | - | TGACAG |
| MA0207.1 | achi | 10.2395 | 1 | 310s_upstream | 278 | 283 | - | TGACAG |
| MA0252.1 | vis | 9.83103 | 1 | 310s_upstream | 278 | 283 | - | TGACAG |
| MA0218.1 | ct | 8.9284 | 1 | 310s_upstream | 296 | 301 | - | TTGAAC |
| MA0210.1 | ara | 6.54466 | 0.954475 | 310s_upstream | 353 | 357 | + | TTACA |
| MA0168.1 | B-H1 | 9.90717 | 0.97745 | 310s_upstream | 371 | 377 | - | TTAAATG |
| MA0170.1 | C15 | 8.82136 | 0.966032 | 310s_upstream | 371 | 377 | - | TTAAATG |

| | | | | | | | | |
|---|---|---|---|---|---|---|---|---|
| MA0199.1 | Optix | 8.73694 | 1 | 310s_upstream | 418 | 422 | - | TGATA |
| MA0204.1 | Six4 | 9.59248 | 1 | 310s_upstream | 417 | 422 | - | TGATAC |
| MA0246.1 | so | 10.3258 | 1 | 310s_upstream | 417 | 422 | - | TGATAC |
| MA0217.1 | caup | 7.88151 | 1 | 310s_upstream | 429 | 433 | - | TAACA |
| MA0210.1 | ara | 7.51791 | 1 | 310s_upstream | 429 | 433 | - | TAACA |
| MA0233.1 | mirr | 7.704 | 0.996821 | 310s_upstream | 429 | 433 | - | TAACA |
| MA0233.1 | mirr | 7.77238 | 1 | 310s_upstream | 441 | 445 | - | AAACA |
| MA0210.1 | ara | 7.42747 | 0.99577 | 310s_upstream | 441 | 445 | - | AAACA |
| MA0210.1 | ara | 6.54466 | 0.954475 | 310s_upstream | 447 | 451 | - | TTACA |
| MA0173.1 | CG11617 | 10.4776 | 0.959704 | 310s_upstream | 446 | 452 | - | TTTACAT |
| MA0199.1 | Optix | 8.73694 | 1 | 310s_upstream | 501 | 505 | - | TGATA |
| MA0237.1 | pan | 9.33623 | 0.968808 | 310s_upstream | 501 | 508 | - | TTTTGATA |
| MA0222.1 | exd | 10.1668 | 0.960086 | 310s_upstream | 501 | 508 | - | TTTTGATA |
| MA0199.1 | Optix | 8.73694 | 1 | 310s_upstream | 510 | 514 | - | TGATA |
| MA0094.1 | Ubx | 6.91669 | 1 | 310s_upstream | 546 | 549 | - | TAAT |
| MA0444.1 | CG34031 | 10.4028 | 0.961359 | 310s_upstream | 544 | 550 | - | TTAATAG |
| MA0094.1 | Ubx | 6.91669 | 1 | 310s_upstream | 548 | 551 | + | TAAT |
| MA0210.1 | ara | 6.45422 | 0.950245 | 310s_upstream | 550 | 554 | + | ATACA |
| MA0094.1 | Ubx | 6.91669 | 1 | 310s_upstream | 569 | 572 | - | TAAT |
| MA0444.1 | CG34031 | 10.4028 | 0.961359 | 310s_upstream | 567 | 573 | - | TTAATAG |
| MA0094.1 | Ubx | 6.91669 | 1 | 310s_upstream | 603 | 606 | - | TAAT |
| MA0094.1 | Ubx | 6.91669 | 1 | 310s_upstream | 638 | 641 | - | TAAT |
| MA0254.1 | vvl | 8.27074 | 1 | 310s_upstream | 655 | 660 | - | TATGCA |
| MA0233.1 | mirr | 7.77238 | 1 | 310s_upstream | 668 | 672 | - | AAACA |
| MA0210.1 | ara | 7.42747 | 0.99577 | 310s_upstream | 668 | 672 | - | AAACA |
| MA0210.1 | ara | 6.54466 | 0.954475 | 310s_upstream | 672 | 676 | + | TTACA |
| MA0210.1 | ara | 6.54466 | 0.954475 | 310s_upstream | 684 | 688 | - | TTACA |
| MA0217.1 | caup | 7.88151 | 1 | 310s_upstream | 686 | 690 | + | TAACA |
| MA0210.1 | ara | 7.51791 | 1 | 310s_upstream | 686 | 690 | + | TAACA |
| MA0233.1 | mirr | 7.704 | 0.996821 | 310s_upstream | 686 | 690 | + | TAACA |
| MA0211.1 | bap | 10.9173 | 0.964051 | 310s_upstream | 691 | 697 | - | TTAAGTA |
| MA0094.1 | Ubx | 6.91669 | 1 | 310s_upstream | 695 | 698 | + | TAAT |
| MA0217.1 | caup | 7.88151 | 1 | 310s_upstream | 714 | 718 | + | TAACA |
| MA0210.1 | ara | 7.51791 | 1 | 310s_upstream | 714 | 718 | + | TAACA |
| MA0233.1 | mirr | 7.704 | 0.996821 | 310s_upstream | 714 | 718 | + | TAACA |
| MA0199.1 | Optix | 8.73694 | 1 | 310s_upstream | 757 | 761 | + | TGATA |
| MA0170.1 | C15 | 8.9577 | 0.971142 | 310s_upstream | 760 | 766 | - | TTAAATA |

| | | | | | | | | |
|---|---|---|---|---|---|---|---|---|
| MA0094.1 | Ubx | 6.91669 | 1 | 310s_upstream | 779 | 782 | + | TAAT |
| MA0190.1 | Gsc | 9.08474 | 0.957136 | 310s_upstream | 779 | 784 | + | TAATCT |
| MA0011.1 | br(var.2) | 9.18293 | 0.955176 | 310s_upstream | 787 | 794 | - | TCCTATTT |
| MA0217.1 | caup | 7.88151 | 1 | 310s_upstream | 802 | 806 | - | TAACA |
| MA0210.1 | ara | 7.51791 | 1 | 310s_upstream | 802 | 806 | - | TAACA |
| MA0233.1 | mirr | 7.704 | 0.996821 | 310s_upstream | 802 | 806 | - | TAACA |
| MA0233.1 | mirr | 7.77238 | 1 | 310s_upstream | 821 | 825 | - | AAACA |
| MA0210.1 | ara | 7.42747 | 0.99577 | 310s_upstream | 821 | 825 | - | AAACA |
| MA0255.1 | z | 10.6504 | 0.951614 | 310s_upstream | 816 | 825 | + | TTGAGTGTTT |
| MA0094.1 | Ubx | 6.91669 | 1 | 310s_upstream | 832 | 835 | - | TAAT |
| MA0457.1 | PHDP | 8.94923 | 0.959385 | 310s_upstream | 830 | 836 | - | CTAATTT |
| MA0094.1 | Ubx | 6.91669 | 1 | 310s_upstream | 849 | 852 | + | TAAT |
| MA0194.1 | Lim1 | 12.2032 | 1 | 310s_upstream | 848 | 854 | + | TTAATTA |
| MA0177.1 | CG18599 | 10.709 | 1 | 310s_upstream | 848 | 854 | + | TTAATTA |
| MA0240.1 | repo | 11.6956 | 1 | 310s_upstream | 848 | 854 | + | TTAATTA |
| MA0179.1 | CG32532 | 10.8324 | 1 | 310s_upstream | 848 | 854 | + | TTAATTA |
| MA0195.1 | Lim3 | 9.90859 | 1 | 310s_upstream | 848 | 854 | + | TTAATTA |
| MA0178.1 | CG32105 | 11.1954 | 1 | 310s_upstream | 848 | 854 | + | TTAATTA |
| MA0176.1 | CG15696 -RA | 10.5589 | 1 | 310s_upstream | 848 | 854 | + | TTAATTA |
| MA0094.1 | Ubx | 6.91669 | 1 | 310s_upstream | 851 | 854 | - | TAAT |
| MA0094.2 | Ubx | 12.0517 | 1 | 310s_upstream | 847 | 854 | + | TTTAATTA |
| MA0236.1 | otp | 10.8604 | 1 | 310s_upstream | 848 | 854 | + | TTAATTA |
| MA0189.1 | E5 | 10.2434 | 1 | 310s_upstream | 848 | 854 | + | TTAATTA |
| MA0170.1 | C15 | 9.72768 | 1 | 310s_upstream | 848 | 854 | + | TTAATTA |
| MA0245.1 | slou | 10.1964 | 1 | 310s_upstream | 848 | 854 | + | TTAATTA |
| MA0167.1 | Awh | 11.159 | 1 | 310s_upstream | 848 | 854 | + | TTAATTA |
| MA0238.1 | pb | 10.7553 | 1 | 310s_upstream | 848 | 854 | + | TTAATTA |
| MA0208.1 | al | 12.1468 | 1 | 310s_upstream | 848 | 854 | - | TAATTAA |
| MA0226.1 | hbn | 10.9717 | 1 | 310s_upstream | 848 | 854 | + | TTAATTA |
| MA0197.1 | nub | 10.741 | 1 | 310s_upstream | 847 | 854 | + | TTTAATTA |
| MA0220.1 | en | 10.7187 | 1 | 310s_upstream | 848 | 854 | + | TTAATTA |
| MA0448.1 | H2.0 | 9.54187 | 1 | 310s_upstream | 848 | 854 | + | TTAATTA |
| MA0257.1 | zen2 | 10.5275 | 1 | 310s_upstream | 848 | 854 | + | TTAATTA |
| MA0172.1 | CG11294 | 11.8189 | 1 | 310s_upstream | 848 | 854 | + | TTAATTA |
| MA0206.1 | abd-A | 11.071 | 1 | 310s_upstream | 848 | 854 | + | TTAATTA |
| MA0251.1 | unpg | 10.8011 | 1 | 310s_upstream | 848 | 854 | + | TTAATTA |

| | | | | | | | | |
|---|---|---|---|---|---|---|---|---|
| MA0181.1 | Vsx1 | 10.8883 | 1 | 310s_upstream | 848 | 854 | + | TTAATTA |
| MA0183.1 | HHEX | 10.9069 | 1 | 310s_upstream | 847 | 854 | + | TTTAATTA |
| MA0232.1 | lbl | 9.18093 | 1 | 310s_upstream | 849 | 854 | + | TAATTA |
| MA0232.1 | lbl | 9.18093 | 1 | 310s_upstream | 849 | 854 | - | TAATTA |
| MA0225.1 | ftz | 10.8544 | 1 | 310s_upstream | 848 | 854 | + | TTAATTA |
| MA0457.1 | PHDP | 9.98515 | 1 | 310s_upstream | 848 | 854 | + | TTAATTA |
| MA0191.1 | HGTX | 10.7393 | 1 | 310s_upstream | 848 | 854 | + | TTAATTA |
| MA0230.1 | lab | 11.2562 | 1 | 310s_upstream | 848 | 854 | + | TTAATTA |
| MA0219.1 | ems | 9.54503 | 0.994755 | 310s_upstream | 848 | 854 | + | TTAATTA |
| MA0231.1 | lbe | 8.33638 | 0.993497 | 310s_upstream | 849 | 854 | + | TAATTA |
| MA0231.1 | lbe | 8.33638 | 0.993497 | 310s_upstream | 849 | 854 | - | TAATTA |
| MA0202.1 | Rx | 10.8659 | 0.992632 | 310s_upstream | 848 | 854 | + | TTAATTA |
| MA0166.1 | Antp | 11.0032 | 0.988327 | 310s_upstream | 848 | 854 | + | TTAATTA |
| MA0214.1 | bsh | 9.46832 | 0.987145 | 310s_upstream | 848 | 854 | + | TTAATTA |
| MA0228.1 | ind | 10.567 | 0.986798 | 310s_upstream | 848 | 854 | + | TTAATTA |
| MA0182.1 | CG4328-RA | 8.83513 | 0.986759 | 310s_upstream | 848 | 854 | + | TTAATTA |
| MA0174.1 | Dbx | 9.51985 | 0.986128 | 310s_upstream | 848 | 854 | + | TTAATTA |
| MA0221.1 | eve | 9.58291 | 0.985684 | 310s_upstream | 848 | 854 | + | TTAATTA |
| MA0209.1 | ap | 10.3463 | 0.983604 | 310s_upstream | 848 | 854 | + | TTAATTA |
| MA0229.1 | inv | 11.5761 | 0.98322 | 310s_upstream | 847 | 854 | + | TTTAATTA |
| MA0184.1 | CG9876 | 10.2467 | 0.98081 | 310s_upstream | 848 | 854 | + | TTAATTA |
| MA0198.1 | OdsH | 10.3598 | 0.979829 | 310s_upstream | 848 | 854 | + | TTAATTA |
| MA0224.1 | exex | 10.536 | 0.977955 | 310s_upstream | 848 | 854 | + | TTAATTA |
| MA0175.1 | lms | 9.85246 | 0.976647 | 310s_upstream | 848 | 854 | + | TTAATTA |
| MA0196.1 | NK7.1 | 9.86415 | 0.976235 | 310s_upstream | 848 | 854 | + | TTAATTA |
| MA0241.1 | ro | 10.5875 | 0.975593 | 310s_upstream | 848 | 854 | + | TTAATTA |
| MA0215.1 | btn | 10.0535 | 0.970571 | 310s_upstream | 848 | 854 | + | TTAATTA |
| MA0200.1 | Pph13 | 9.7241 | 0.967021 | 310s_upstream | 848 | 854 | + | TTAATTA |
| MA0250.1 | unc-4 | 10.1978 | 0.960935 | 310s_upstream | 848 | 854 | + | TTAATTA |
| MA0203.1 | Scr | 10.1754 | 0.958553 | 310s_upstream | 848 | 854 | + | TTAATTA |
| MA0200.1 | Pph13 | 10.1978 | 0.98585 | 310s_upstream | 849 | 855 | - | ATAATTA |
| MA0182.1 | CG4328-RA | 8.73106 | 0.98287 | 310s_upstream | 849 | 855 | - | ATAATTA |
| MA0457.1 | PHDP | 9.31039 | 0.973545 | 310s_upstream | 849 | 855 | - | ATAATTA |
| MA0187.1 | Dll | 9.20701 | 0.967551 | 310s_upstream | 849 | 855 | + | TAATTAT |
| MA0177.1 | CG18599 | 9.6095 | 0.960669 | 310s_upstream | 849 | 855 | - | ATAATTA |
| MA0238.1 | pb | 9.5289 | 0.957237 | 310s_upstream | 849 | 855 | - | ATAATTA |

| | | | | | | | | |
|---|---|---|---|---|---|---|---|---|
| MA0195.1 | Lim3 | 8.76182 | 0.955193 | 310s_upstream | 849 | 855 | - | ATAATTA |
| MA0191.1 | HGTX | 9.4958 | 0.952941 | 310s_upstream | 849 | 855 | - | ATAATTA |
| MA0199.1 | Optix | 8.73694 | 1 | 310s_upstream | 875 | 879 | + | TGATA |
| MA0233.1 | mirr | 7.77238 | 1 | 310s_upstream | 888 | 892 | - | AAACA |
| MA0210.1 | ara | 7.42747 | 0.99577 | 310s_upstream | 888 | 892 | - | AAACA |
| MA0233.1 | mirr | 7.77238 | 1 | 310s_upstream | 894 | 898 | - | AAACA |
| MA0210.1 | ara | 7.42747 | 0.99577 | 310s_upstream | 894 | 898 | - | AAACA |
| MA0233.1 | mirr | 7.77238 | 1 | 310s_upstream | 898 | 902 | - | AAACA |
| MA0210.1 | ara | 7.42747 | 0.99577 | 310s_upstream | 898 | 902 | - | AAACA |
| MA0210.1 | ara | 6.54466 | 0.954475 | 310s_upstream | 902 | 906 | + | TTACA |
| MA0217.1 | caup | 7.88151 | 1 | 310s_upstream | 945 | 949 | + | TAACA |
| MA0210.1 | ara | 7.51791 | 1 | 310s_upstream | 945 | 949 | + | TAACA |
| MA0233.1 | mirr | 7.704 | 0.996821 | 310s_upstream | 945 | 949 | + | TAACA |
| MA0185.1 | Deaf1 | 6.96107 | 0.958761 | 310s_upstream | 949 | 954 | - | TTCGGT |
| MA0199.1 | Optix | 8.73694 | 1 | 310s_upstream | 973 | 977 | + | TGATA |
| MA0094.1 | Ubx | 6.91669 | 1 | 310s_upstream | 996 | 999 | + | TAAT |
| MA0190.1 | Gsc | 9.08474 | 0.957136 | 310s_upstream | 996 | 1001 | + | TAATCT |
| MA0011.1 | br(var.2) | 10.2163 | 1 | 310s_upstream | 1007 | 1014 | - | TACTATTT |
| MA0217.1 | caup | 7.88151 | 1 | 310s_upstream | 1020 | 1024 | - | TAACA |
| MA0210.1 | ara | 7.51791 | 1 | 310s_upstream | 1020 | 1024 | - | TAACA |
| MA0233.1 | mirr | 7.704 | 0.996821 | 310s_upstream | 1020 | 1024 | - | TAACA |
| MA0233.1 | mirr | 7.77238 | 1 | 310s_upstream | 1037 | 1041 | - | AAACA |
| MA0210.1 | ara | 7.42747 | 0.99577 | 310s_upstream | 1037 | 1041 | - | AAACA |
| MA0210.1 | ara | 6.54466 | 0.954475 | 310s_upstream | 1043 | 1047 | + | TTACA |
| MA0210.1 | ara | 6.54466 | 0.954475 | 310s_upstream | 1084 | 1088 | + | TTACA |
| MA0049.1 | hb | 11.613 | 0.962703 | 310s_upstream | 1105 | 1114 | + | GAAAAAAAAA |
| MA0233.1 | mirr | 7.77238 | 1 | 310s_upstream | 1113 | 1117 | + | AAACA |
| MA0210.1 | ara | 7.42747 | 0.99577 | 310s_upstream | 1113 | 1117 | + | AAACA |
| MA0199.1 | Optix | 8.73694 | 1 | 310s_upstream | 1126 | 1130 | + | TGATA |
| MA0011.1 | br(var.2) | 10.2163 | 1 | 310s_upstream | 1151 | 1158 | - | TACTATTT |
| MA0233.1 | mirr | 7.77238 | 1 | 310s_upstream | 1187 | 1191 | - | AAACA |

| | | | | | | | | |
|---|---|---|---|---|---|---|---|---|
| MA0210.1 | ara | 7.42747 | 0.99577 | 310s_upstream | 1187 | 1191 | - | AAACA |
| MA0094.1 | Ubx | 6.91669 | 1 | 310s_upstream | 1195 | 1198 | - | TAAT |
| MA0448.1 | H2.0 | 8.56383 | 0.962773 | 310s_upstream | 1196 | 1202 | - | TTTATAA |
| MA0094.1 | Ubx | 6.91669 | 1 | 310s_upstream | 1228 | 1231 | - | TAAT |
| MA0232.1 | lbl | 8.49402 | 0.97079 | 310s_upstream | 1226 | 1231 | - | TAATGA |
| MA0219.1 | ems | 9.681 | 1 | 310s_upstream | 1226 | 1232 | - | TTAATGA |
| MA0166.1 | Antp | 11.3252 | 1 | 310s_upstream | 1226 | 1232 | - | TTAATGA |
| MA0186.1 | Dfd | 11.575 | 1 | 310s_upstream | 1226 | 1232 | - | TTAATGA |
| MA0215.1 | btn | 10.8699 | 1 | 310s_upstream | 1226 | 1232 | - | TTAATGA |
| MA0203.1 | Scr | 11.3978 | 1 | 310s_upstream | 1226 | 1232 | - | TTAATGA |
| MA0225.1 | ftz | 10.8544 | 1 | 310s_upstream | 1226 | 1232 | - | TTAATGA |
| MA0221.1 | eve | 9.84186 | 0.995087 | 310s_upstream | 1226 | 1232 | - | TTAATGA |
| MA0238.1 | pb | 10.5367 | 0.992378 | 310s_upstream | 1226 | 1232 | - | TTAATGA |
| MA0189.1 | E5 | 9.82099 | 0.985257 | 310s_upstream | 1226 | 1232 | - | TTAATGA |
| MA0214.1 | bsh | 9.27568 | 0.979453 | 310s_upstream | 1226 | 1232 | - | TTAATGA |
| MA0257.1 | zen2 | 9.91196 | 0.977237 | 310s_upstream | 1226 | 1232 | - | TTAATGA |
| MA0230.1 | lab | 10.6041 | 0.976296 | 310s_upstream | 1226 | 1232 | - | TTAATGA |
| MA0170.1 | C15 | 9.08226 | 0.97581 | 310s_upstream | 1226 | 1232 | - | TTAATGA |
| MA0448.1 | H2.0 | 8.88548 | 0.975016 | 310s_upstream | 1226 | 1232 | - | TTAATGA |
| MA0256.1 | zen | 10.3537 | 0.973049 | 310s_upstream | 1226 | 1232 | - | TTAATGA |
| MA0174.1 | Dbx | 9.15728 | 0.972255 | 310s_upstream | 1226 | 1232 | - | TTAATGA |
| MA0206.1 | abd-A | 10.1836 | 0.968084 | 310s_upstream | 1226 | 1232 | - | TTAATGA |
| MA0177.1 | CG18599 | 9.80997 | 0.96784 | 310s_upstream | 1226 | 1232 | - | TTAATGA |
| MA0228.1 | ind | 9.93778 | 0.96457 | 310s_upstream | 1226 | 1232 | - | TTAATGA |
| MA0191.1 | HGTX | 9.7513 | 0.962611 | 310s_upstream | 1226 | 1232 | - | TTAATGA |
| MA0245.1 | slou | 8.89884 | 0.950051 | 310s_upstream | 1226 | 1232 | - | TTAATGA |
| MA0197.1 | nub | 9.97791 | 0.971788 | 310s_upstream | 1226 | 1233 | - | TTTAATGA |

| | | | | | | | | |
|---|---|---|---|---|---|---|---|---|
| MA0094.2 | Ubx | 10.965 | 0.963518 | 310s_upstream | 1226 | 1233 | - | TTTAATGA |
| MA0210.1 | ara | 6.45422 | 0.950245 | 310s_upstream | 1242 | 1246 | + | ATACA |
| MA0170.1 | C15 | 8.9577 | 0.971142 | 310s_upstream | 1247 | 1253 | - | TTAAATA |
| MA0211.1 | bap | 11.9861 | 1 | 310s_upstream | 1250 | 1256 | + | TTAAGTG |
| MA0217.1 | caup | 7.88151 | 1 | 310s_upstream | 1255 | 1259 | - | TAACA |
| MA0210.1 | ara | 7.51791 | 1 | 310s_upstream | 1255 | 1259 | - | TAACA |
| MA0233.1 | mirr | 7.704 | 0.996821 | 310s_upstream | 1255 | 1259 | - | TAACA |
| MA0210.1 | ara | 6.45422 | 0.950245 | 310s_upstream | 1270 | 1274 | - | ATACA |
| MA0254.1 | vvl | 8.04232 | 0.989087 | 310s_upstream | 1314 | 1319 | - | TATTCA |
| MA0185.1 | Deaf1 | 7.74071 | 1 | 310s_upstream | 1328 | 1333 | - | TTCGGG |
| MA0233.1 | mirr | 7.77238 | 1 | 310s_upstream | 1362 | 1366 | - | AAACA |
| MA0210.1 | ara | 7.42747 | 0.99577 | 310s_upstream | 1362 | 1366 | - | AAACA |
| MA0227.1 | hth | 9.78892 | 1 | 310s_upstream | 1386 | 1391 | - | TGACAG |
| MA0207.1 | achi | 10.2395 | 1 | 310s_upstream | 1386 | 1391 | - | TGACAG |
| MA0252.1 | vis | 9.83103 | 1 | 310s_upstream | 1386 | 1391 | - | TGACAG |
| MA0185.1 | Deaf1 | 7.74071 | 1 | 310s_upstream | 1444 | 1449 | + | TTCGGG |
